# Methodology for Impedance Spectroscopy of Photovoltaic Modules Using a Power Converter

**DOI:** 10.3390/s26010161

**Published:** 2025-12-25

**Authors:** Diego Alejandro Herrera-Jaramillo, Juan David Bastidas-Rodríguez, Carlos Andrés Ramos-Paja, Carlos Pavon-Vargas, Luis E. Garcia-Marrero, Sergio Ignacio Serna-Garcés

**Affiliations:** 1 Facultad de Ingenierías, Institución Universitaria ITM, Medellín 050036, Colombia; 2 Facultad de Ingeniería y Arquitectura, Universidad Nacional de Colombia, Manizales 170003, Colombia; jubastidasr@unal.edu.co; 3 Facultad de Minas, Universidad Nacional de Colombia, Medellín 050043, Colombia; caramosp@unal.edu.co; 4 Dipartimento di Ingegneria dell’Informazione ed Elettrica e Matematica Applicata/DIEM, University of Salerno, 84084 Fisciano, Italy; cpavonvargas@unisa.it (C.P.-V.); lgarciamarrero@unisa.it (L.E.G.-M.); 5 Departamento de Electrónica y Telecomunicaciones, Institución Universitaria ITM, Medellín 050013, Colombia; sergioserna@itm.edu.co

**Keywords:** impedance spectroscopy, photovoltaic modules, synchronous boost converter

## Abstract

Impedance Spectroscopy (IS) is widely used to analyze the dynamic behavior and degradation of electrochemical systems such as batteries. IS has also been successfully applied to study the performance and degradation mechanisms of photovoltaic (PV) devices. Traditionally, IS is performed with Frequency Response Analyzers (FRA), which apply small-signal perturbations and measure the impedance response of the system. However, those instruments are costly and not suitable for in situ diagnostics. This work proposes a methodology to perform IS measurements on PV systems using a power converter, thereby eliminating the need for external specialized equipment. The proposed approach includes a theoretical analysis of the converter dynamics to derive an expression for the duty cycle amplitude, which is required to maintain a constant perturbation magnitude across a range of frequencies. The methodology is experimentally validated using a synchronous Boost converter connected to a PV panel and controlled by a Texas Instruments F28379D digital signal processor (DSP), which injects the perturbation signal in the converter’s duty cycle. Moreover, the voltage and current measurements are performed with an oscilloscope. The results demonstrate that the proposed converter-based IS method accurately reproduces the impedance spectra obtained with a commercial FRA, confirming its feasibility as a low-cost, flexible, and scalable solution for PV impedance characterization and diagnostics.

## 1. Introduction

Photovoltaic (PV) energy generation continues to expand rapidly, driven by both mature silicon-based technologies and the ongoing development of emerging alternatives such as perovskite solar cells [1,2]. While novel technologies are expected to play an important role in future PV deployments, silicon-based modules currently dominate utility-scale solar plants worldwide [3]. As a result, ensuring the performance, reliability, and lifetime of silicon PV installations has become a critical challenge, particularly at the scale of large solar plants. This has intensified the demand for scalable and cost-effective diagnostic techniques that can be applied in a massive manner without disrupting energy production.

Conventional diagnostic approaches, such as infrared thermography and current-voltage (I-V) curve tracing, remain widely used in current PV systems but present important limitations. Thermographic inspections enable the visual identification of defects such as hot spots or faulty interconnections; however, their effectiveness strongly depends on the operator’s expertise, proper equipment calibration, and specialized instrumentation, which increases inspection costs. Similarly, I-V curve measurements provide valuable electrical information but typically require module disconnection or additional hardware, leading to production interruptions. Moreover, both techniques often rely on computationally intensive post-processing algorithms for defect classification, further limiting their suitability for large-scale deployment. In this context, impedance spectroscopy (IS) has emerged as a powerful diagnostic technique capable of providing detailed insight into the electrochemical and electrical behavior of devices and materials [4,5]. By applying a small periodic excitation over a defined frequency range and measuring the system response, IS enables the extraction of frequency-dependent impedance characteristics [6]. Depending on the excitation mode, current perturbation (galvanostatic) or voltage perturbation (potentiostatic), the resulting spectra reveal charge-transfer dynamics, resistive and capacitive effects, and various degradation mechanisms. The ability to decouple the previous phenomena has become IS, a widely adopted tool in electrochemistry, battery diagnostics, fuel-cell analysis, and, more recently, has also increased its relevance in PV systems.

Within the PV field, IS has been used to analyze module degradation and aging [7]. Traditionally, these measurements are performed indoors using specialized frequency response analyzers (FRA) [8]. However, removing the device under test (DUT) from its operational environment increases cost and time while interrupting energy production. To address this limitation, several recent studies have explored outdoor and online IS implementations that account for real environmental variations such as irradiance, temperature, and humidity [9]. A significant advance in this direction has been the use of power converters (PCs) to perform IS directly on PV modules [10,11]. In those approaches, a sinusoidal perturbation is injected into the PV current by modulating the converter’s duty cycle around a steady operating point (galvanostatic mode), while the resulting voltage response is measured to compute the impedance. This enables in situ diagnostics without interrupting power generation.

Building on those foundations, several works have demonstrated the applicability of IS to practical PV scenarios. For instance, ref. [12] combined experimental and simulation-based analysis to evaluate partial shading effects in series-connected panels under outdoor conditions, demonstrating that IS can detect the mismatching phenomena that remain hidden in conventional I-V measurements. Their improved dynamic model, based on constant phase elements (CPEs) and validated through SPICE simulations, provided detailed characterization of non-uniform operating conditions without disturbing PV operation.

Complementarily, ref. [13] proposed a methodology to characterize the dynamic behavior of PV cells under controlled laboratory conditions using IS, integrating the diode’s dynamic capacitance and conductance into an enhanced single-diode model (SDM) based on the Berkeley SPICE representation [14]. Although reducing the need for extensive I-V sweeps, this approach remained limited to single-cell experiments and fixed environmental conditions, requiring new approaches for module-level behavior under realistic mismatch scenarios. Additional validation of IS for partial shading detection was presented in [15], where controlled shading patterns were correlated with electrical and thermal responses obtained from I-V and infrared measurements, confirming IS as a fast and reliable diagnostic tool. Nevertheless, those studies relied on external FRA equipment and fixed-frequency excitation, thus restricting their suitability for scalable and real-time field diagnostics.

In parallel, ref. [16] introduced a self-adaptive methodology for real-time parameter identification in PV modules using an enhanced SDM and adaptive filters. Although not directly implementing IS, this framework is aimed to in situ dynamic characterization, suggesting that integrating converter-based IS into such adaptive schemes could produce a more comprehensive diagnostic platform.

Therefore, recent research has been focused on enabling IS measurements directly through power converters, avoiding the cost, size, and operational interruption associated with standalone FRAs. Multiple works have demonstrated the feasibility of converter-based IS under real operating conditions by injecting either voltage or current perturbations through the converter control loop. Adaptive open-loop techniques have been proposed to mitigate resonance issues and extend the usable frequency range [17], while cooperative maximum power point tracking (MPPT) and IS controllers have enabled accurate impedance measurements with minimal impact on energy production [18]. These contributions highlight the potential of converter-assisted IS, but also reveal significant control complexity and a strong dependence on detailed converter models.

Alternative excitation strategies have been explored to reduce hardware constraints. Some authors have proposed using the natural inductor current ripple of a boost converter as the excitation signal [19], and others have used the wide-bandgap SiC semiconductors to achieve the high frequencies required for PV impedances [10]. Although these approaches reduce measurement time or enable higher bandwidth, they remain bounded by specific hardware conditions (e.g., high irradiance or high switching frequencies). Moreover, those solutions do not provide a systematic method to ensure constant perturbation amplitude across wide frequency ranges, which is essential for accurate impedance reconstruction.

Other efforts have focused on dynamic PV parameter extraction using converter-based IS. In this way, panel-level equivalent circuit models and health-feature extraction methods have been proposed for low-frequency IS (1 Hz–2 kHz) [20]. Instead, other studies have evaluated the validity of AC equivalent models under multiple operating conditions, establishing guidelines for perturbation selection based on total harmonic distortion and Kramers-Kronig criteria [7]. Fast IS acquisition using broadband excitation has also been demonstrated experimentally [11]. However, those studies typically rely on external FRA equipment for validation, assume fixed-amplitude excitation, or restrict the usable frequency range to low-frequency dynamics where converter limitations are less dominant.

In conclusion, the previous literature review confirms the feasibility of performing IS using power converters, but reveals a clear gap: none of the reviewed works provides a practical methodology to define the duty-cycle perturbation amplitude required to guarantee a controlled excitation magnitude at the PV terminals, across a wide frequency range, and accounting for the intrinsic dynamics of the converter. This limitation compromises measurement accuracy, especially at medium and high frequencies where converter behavior significantly shapes the injected signal. Moreover, such a methodology must be experimentally tested to evaluate its effectiveness under practical constraints, including parasitic effects and dead-times of the MOSFETs activation, among other real operation conditions.

Considering those gaps, this work proposes a methodology for performing IS in PV systems using a power converter, and without external specialized FRA equipment. The approach includes a theoretical analysis of the converter dynamics to derive the duty-cycle amplitude necessary to impose a constant perturbation magnitude across a wide frequency range. Experimental validation was conducted using a synchronous boost converter connected to a PV module, which is controlled with an F28379D digital signal processor (DSP) (Texas Instruments, Dallas, TX, USA) to inject the perturbation signal, while PV voltage and current were measured with an oscilloscope. These data were processed using fast Fourier transform (FFT) analysis to compute the impedance spectrum. The results show strong agreement with commercial FRA-based measurements, validating the proposed method as a low-cost, flexible, and accurate alternative for PV impedance characterization.

## 2. Proposed System Modeling for Impedance Spectroscopy

**Figure 1:**
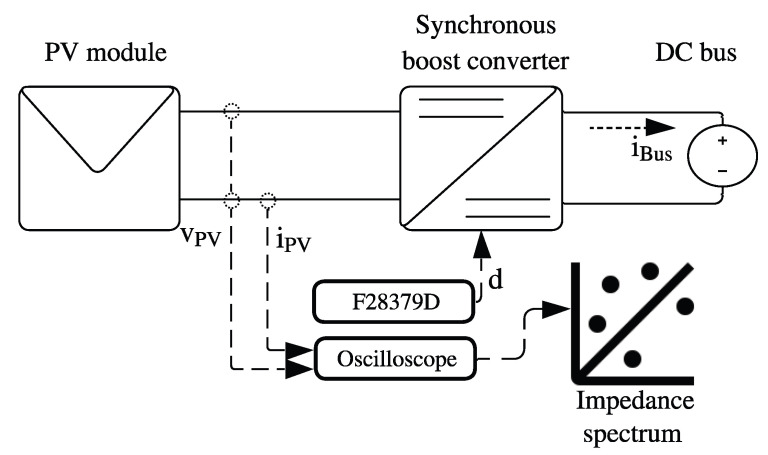
Proposed structure to perform IS on a PV module.

The proposed structure used to perform IS measurements on a PV module, by using the power converter, is depicted in Figure 1. The PV module is connected to a DC bus through a synchronous Boost converter, where the DC bus represents the input of an inverter or the bus of a DC microgrid. The Texas Instruments F28379D controls the Boost converter, while the voltage and current signals from the PV module are measured using an oscilloscope. Finally, the recorded data are processed offline to calculate the impedance at each disturbance frequency and generate the impedance spectrum. The following subsections describe the model of the proposed system.

### 2.1. PV Module Modelling

PV modules are usually represented by the SDM, which is a static model used to reproduce the PV module’s I-V curve. Such a model includes the current source ($I_{ph}$) that represents the photocurrent generated by the photovoltaic effect, the resistor $R_{s}$ that models the ohmic losses in the semiconductor material, interconnections, and contacts, the resistor $R_{sh}$ that represents the leakage currents through the p-n junction and material imperfections, and a diode that represent the static nonlinearities of the PV cells.

**Figure 2:**
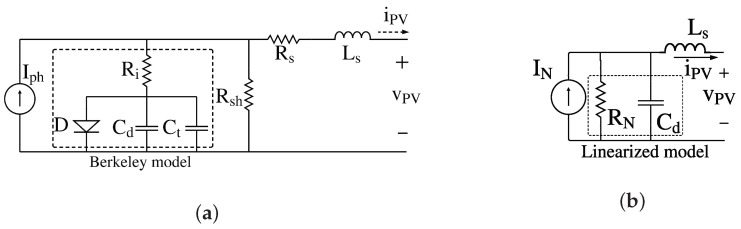
Dynamic approach based on the SDM model. (**a**) Dynamic approach based on the SDM model. (**b**) Linearized model around an operating point.

However, the SDM does not consider the PV module small-signal dynamics; then, the SDM can be extended by including an inductance ($L_{s}$) in series with $R_{s}$ to represent the parasitic inductance of the module’s cabling and connectors. Moreover, the model can be inproved by representing the diode of the SDM with the Berkeley diode model [13,14], which captures the diode’s static and dynamic behavior through four elements (see Figure 2a): a diode with the exponential model (*D*) that reproduces the nonlinear static behavior of the PV cells, the resistance $R_{i}$ that accounts for the carrier transport and recombination-related resistive effects within the junction, the depletion capacitance ($C_{t}$), which dominates under reverse bias, and the diffusion capacitance ($C_{d}$), which becomes significant under forward bias. Moreover, in Figure 2a, $v_{PV}$ and $i_{PV}$ denote the voltage and current of the PV module, respectively.

While the detailed equivalent circuit provides an accurate representation of the PV module’s nonlinear and dynamic behavior, its complexity makes direct analytical treatment impractical, particularly for impedance-based analysis. To facilitate the application of IS, the system can be linearized around a steady-state operating point using its small-signal model, assuming that the perturbation amplitude remains small (on the order of milliamperes and millivolts), which is the case in IS applications [9].

For the large-signal static model, the capacitors ($C_{d}$ and $C_{t}$) and the inductor ($L_{s}$) behave as open-circuit and short-circuit, respectively; hence, the PV module can be represented by $I_{ph}$, $R_{i}$, *D*, $R_{sh}$, and $R_{s}$. In this representation, the nonlinear behavior of the module is dominated by the diode, whose current is described by the Shockley Equation (1),

(1) $$I_{D}=I_{\mathrm{sat}}\left[\exp\left(\frac{V_{D}}{\eta V_{T}}\right)-1\right],$$

where $I_{\mathrm{sat}}$ is the reverse saturation current, η is the ideality factor, and $V_{T}=kT/q$ is the thermal voltage (*k* is the Boltzmann constant, *q* the electron charge, and *T* the absolute temperature). These parameters govern the curvature and slope of the I-V characteristic, therefore those parameters define the operating point of the module. Such a circuit can be used to reproduce the PV module’s I-V curve and determine the operating point ($V_{Q}$, $I_{Q}$) where the IS is measured. The presence of (1) in the model clarifies the nonlinear contribution of the diode that underlies both the static model and the subsequent derivation of the Norton equivalent.

**Figure 3:**
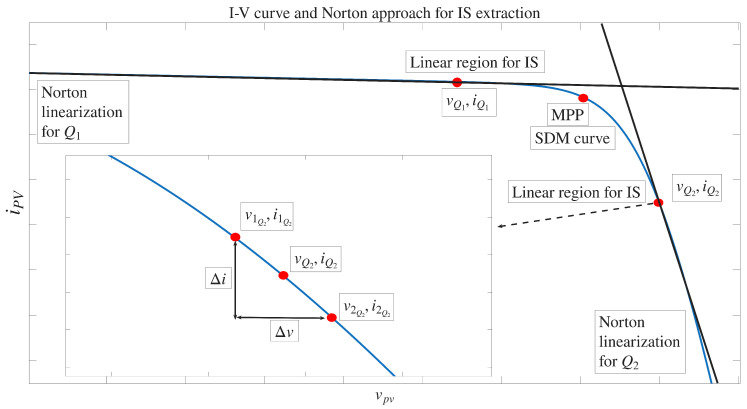
SDM linearization around a point Q.

Once the operating point $\left(V_{Q},I_{Q}\right)$ is established, the Norton equivalent circuit around $\left(V_{Q},I_{Q}\right)$ can be obtained using (2) and (3), where $\left(V_{1},I_{1}\right)$ and $\left(V_{2},I_{2}\right)$ are two points on the I-V curve to the left and right of $\left(V_{Q},I_{Q}\right)$, respectively, as shown in Figure 3.

(2) $$\begin{array}{ccc} R_{N} & = & -\frac{V_{2}-V_{1}}{I_{2}-I_{1}} \end{array}$$

(3) $$\begin{array}{ccc} I_{N} & = & I_{1}+\frac{V_{1}}{R_{N}} \end{array}$$

The small-signal static and dynamic linearized model of the PV module is obtained by including $C_{d}$ in parallel with the Norton resistance ($R_{N}$), and by accounting for $L_{s}$ that appears in series with the Norton equivalent (see Figure 2b). Only the diffusion capacitance is included because it is the dominant capacitance when the PV module is forward biased [21]. In addition, $C_{d}$ depends on the operating point voltage $V_{Q}$ according to (4), where $T_{T}$ is the forward transit time. The remaining diode parameters ($I_{s}$, η, and $V_{T}$) correspond to those already defined in the Shockley Equation (1).

(4) $$\begin{array}{c} C_{d}=\frac{T_{T}I_{s}}{\eta V_{t}}\exp\left(\frac{V_{Q}}{\eta V_{t}}\right) \end{array}$$

Finally, the equivalent linearized model of the PV module, shown in Figure 2b, is used to reproduce the small-signal static and dynamic behaviors of the PV module around a given operating point $\left(V_{Q},I_{Q}\right)$.

### 2.2. Modelling of the Power Conversion System

**Figure 4:**
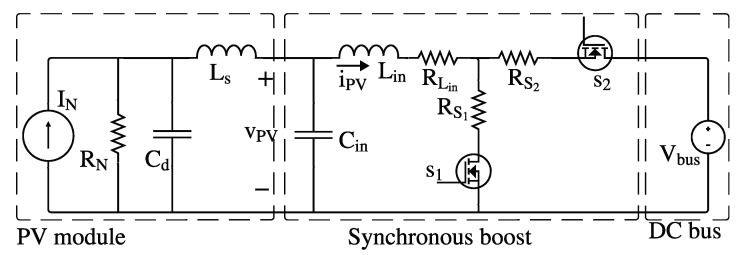
Small-signal dynamic model of the PV module coupled with a synchronous Boost converter and a DC bus.

The small-signal equivalent circuit of the PV module connected to a DC bus through a Boost converter is reported in Figure 4, where each element of the system is defined with dashed lines. In such a circuit, $C_{in}$ and $L_{in}$ are the converter’s input capacitance and inductance, respectively, $R_{Lin}$ is the inductor’s equivalent series resistance, $S_{1}$ and $S_{2}$ are the MOSFETs used to implement the synchronous converter, $R_{S1}$ and $R_{S2}$ are the MOSFETs’ on resistances and $V_{bus}$ is the voltage of the DC bus.

In such a circuit, $C_{d}\ll C_{in}$ and $L_{s}\ll L_{in}$, hence the dynamic response of the system is dominated by the converter’s dynamics and the PV module is usually represented by static equivalent circuits, like the SDM. Therefore, $C_{d}$ and $L_{s}$ can be neglected in the analysis of the dynamic response of the system presented in Figure 4.

For a galvanostatic IS, the control objective is to set a constant amplitude of the sinusoidal perturbation induced in the inductor current ($i_{PV}$) for any excitation frequency ($f_{IS}$) and at any operating point, as reported in [22]. To achieve this, the converter duty cycle must be modulated to generate a sinusoidal perturbation that produces the desired variation in $i_{PV}$, thus compensating the impedance variation on the PV module and power converter.

In the average model of the Boost converter, the duty cycle is expressed as a time-varying quantity d(t) that consists of a steady-state component and a small-signal perturbation, as shown in (5). The constant component $d_{DC}$ defines the operating point (*Q*), while the sinusoidal term $d_{IS}\sin\left(\omega t\right)$ represents the perturbation used to perform the impedance spectroscopy. This small-signal excitation produces a corresponding sinusoidal variation in the PV current with amplitude $\left|I_{PV}\left(j\omega \right)\right|$.

(5) $$d\left(t\right)=d_{DC}+d_{IS}\sin\left(\omega t\right)$$

Starting from Figure 4, the two state equations of the averaged model are given in (6) and (7), where $v_{PV}$ is the voltage across $C_{in}$ and $i_{PV}$ is the current through $L_{in}$. Assuming that the switches $S_{1}$ and $S_{2}$ are identical, the corresponding resistances can be defined as $R_{S}=R_{S_{1}}=R_{S_{2}}$. Consequently, the power losses associated with $L_{in}$, $S_{1}$, and $S_{2}$ can be simplified as given in (8), resulting in the total equivalent resistance ($R_{T}$) also reported in (8). Thus, $R_{T}$ is incorporated into the inductor voltage Equation (6) to represent the power losses.

(6) $$L_{in}\frac{d}{dt}i_{PV}=v_{C_{in}}-i_{PV}\cdot R_{T}-V_{bus}\cdot \left(1-d\left(t\right)\right)$$

(7) $$C_{in}\frac{d}{dt}v_{PV}=I_{N}-\frac{v_{PV}}{R_{N}}-i_{PV}$$

(8) $$R_{T}=R_{L_{in}}+R_{S}d_{DC}+R_{S}\left(1-d_{DC}\right)=R_{L_{in}}+R_{S}$$

Solving (7) for $I_{PV}$ and substituting into (6), with $I_{N}=0\;A$, leads to the Laplace domain Equation (9), which represents the variation of the PV current with respect to the duty-cycle perturbation.

(9) $$I_{PV}\left(s\right)=\frac{V_{bus}\left(R_{N}C_{in}\cdot s+1\right)}{R_{N}L_{in}C_{in}\cdot s^{2}+\left(L_{in}+R_{T}R_{N}C_{in}\right)s+R_{N}+R_{T}}d_{IS}\left(s\right)$$

For sinusoidal perturbations s=jω where $\omega =2\pi f_{IS}$. Substituting s=jω into (9) gives (10), from which the frequency response of $i_{PV}$ to a sinusoidal d(t) is calculated. The resulting amplitude of $I_{PV}\left(j\omega \right)$ is reported in (11).

(10) $$I_{PV}\left(j\omega \right)=\frac{V_{bus}\left(R_{N}C_{in}\left(j\omega \right)+1\right)}{R_{N}L_{in}C_{in}{\left(j\omega \right)}^{2}+\left(L_{in}+R_{T}R_{N}C_{in}\right)\left(j\omega \right)+R_{N}+R_{T}}d_{IS}\left(j\omega \right)$$

(11) $$\left|I_{PV}\left(j\omega \right)\right|=\frac{V_{bus}\sqrt{1+{\left(R_{N}C_{in}\omega \right)}^{2}}}{\sqrt{{\left(R_{N}+R_{T}-R_{N}L_{in}C_{in}\omega^{2}\right)}^{2}+{\left((L_{in}+R_{T}R_{N}C_{in})\omega \right)}^{2}}}\left|d_{IS}\left(j\omega \right)\right|$$

Finally, solving for $\left|d_{IS}\left(j\omega \right)\right|$ in (11) leads to (12), which defines the amplitude of the duty-cycle perturbation required to produce a desired PV current amplitude at a given frequency.

(12) $$|d_{IS}\left(j\omega \right)|=\frac{|I_{PV}\left(j\omega \right)|}{\frac{V_{bus}\sqrt{1+{\left(R_{N}C_{in}\omega \right)}^{2}}}{\sqrt{{\left(R_{N}+R_{T}-R_{N}L_{in}C_{in}\omega^{2}\right)}^{2}+{\left((L_{in}+R_{T}R_{N}C_{in})\omega \right)}^{2}}}}$$

The instantaneous PV current $i_{PV}\left(t\right)$ can therefore be expressed as (13), where $i_{DC}$ is the average current at the operating point *Q* due to $d_{DC}$, $i_{SW}\left(t\right)$ represents the high-frequency switching ripple, and $i_{IS}\left(t\right)$ is the small-signal component defined in (14). In this expression, $i_{p}=\left|I_{PV}\left(j\omega \right)\right|$ denotes the amplitude of the sinusoidal perturbation, which remains constant for all test frequencies, and it is typically in the milliampere range.

(13) $$\begin{array}{c} i_{PV}\left(t\right)=i_{DC}+i_{SW}\left(t\right)+i_{IS}\left(t\right) \end{array}$$

(14) $$\begin{array}{c} i_{IS}\left(t\right)=i_{p}\sin\left(\omega t\right) \end{array}$$

Similarly, the PV voltage $v_{PV}\left(t\right)$ can be described as in (15), where $v_{DC}$ is the average voltage at operating point *Q*, $v_{SW}\left(t\right)$ is the high-frequency ripple due to the MOSFETs’ switching, and $v_{IS}\left(t\right)$ is the sinusoidal response of the PV voltage to $i_{IS}\left(t\right)$, defined in (16). In (16), $v_{p}$ is the peak amplitude of the voltage perturbation and θ is the phase shift taking $i_{IS}\left(t\right)$ as reference.

(15) $$\begin{array}{c} v_{PV}\left(t\right)=v_{DC}+v_{SW}\left(t\right)+v_{IS}\left(t\right) \end{array}$$

(16) $$\begin{array}{c} v_{IS}\left(t\right)=v_{p}\sin\left(\omega t+\theta \right) \end{array}$$

#### Impedance Calculation

The impedance of the PV module is obtained from the small-signal perturbations injected and measured at its terminals. This measurement location is essential to ensure that the computed impedance corresponds exclusively to the PV module and is not affected by the internal dynamics of the DC-DC converter. In this configuration, the converter only provides the controlled excitation, while the PV module is the single element within the impedance-measurement boundary.

For each selected excitation frequency $f_{is}$, a sinusoidal current perturbation is generated according to the small-signal component $i_{IS}\left(t\right)$ defined in (14). The resulting instantaneous PV current and voltage waveforms, $i_{PV}\left(t\right)$ and $v_{PV}\left(t\right)$, contain the DC operating point, the switching ripple, and the small-signal IS component, as expressed in (13) and (15). The switching-frequency components $i_{SW}\left(t\right)$ and $v_{SW}\left(t\right)$ can be disregarded for IS purposes since the excitation frequency satisfies $f_{is}\ll f_{SW}$.

The IS components of interest, $i_{IS}\left(t\right)$ and $v_{IS}\left(t\right)$, were already defined in (14) and (16), respectively. To obtain their complex phasors, the measured signals $i_{PV}\left(t\right)$ and $v_{PV}\left(t\right)$ are processed using a Fast Fourier Transform (FFT). The complex coefficient at the excitation frequency provides the magnitude and phase of the small-signal components given in (17), where $i_{p}$ and $v_{p}$ correspond to the peak amplitudes defined in (14) and (16), and θ is the phase shift between voltage and current waveforms. Finally, the impedance of the PV module at frequency $f_{is}$ is computed using (18).

(17) $$\begin{array}{c} {\hat{I}}_{IS}\left(f_{is}\right)=i_{p}∠0,\;{\hat{V}}_{IS}\left(f_{is}\right)=v_{p}∠\theta \end{array}$$

(18) $$\begin{array}{c} Z\left(f_{is}\right)=\frac{{\hat{V}}_{IS}\left(f_{is}\right)}{{\hat{I}}_{IS}\left(f_{is}\right)} \end{array}$$

This procedure provides the impedance for a single test frequency. To obtain the complete IS spectrum, the same process is repeated for a set of *N* frequencies within the desired range. The resulting values of Z(f) can be represented using Nyquist and Bode plots to analyze the frequency-dependent behavior of the PV module.

## 3. Power Converter Considerations for IS

In order to perform IS on PV modules using a power electronics interface, the configuration and operation of the converter are fundamental for ensuring both the accuracy of the perturbation and the reliability of the impedance calculation. The following subsections present the main implementation aspects and limitations in this context.

### 3.1. Duty Cycle Operation for Perturbation

The sinusoidal perturbation is generated by modulating the duty cycle of the synchronous Boost converter. Following Equation (5), a fixed value of the duty cycle component $d_{DC}$ is first defined, placing the operating point of the PV module to the right of the Maximum Power Point (MPP). The perturbation at a chosen frequency $f_{IS}$ is then introduced by calculating the perturbation term $d_{IS}$ using Equation (12).

As a result of applying Equations (5) and (12), $i_{PV}\left(t\right)$ in Equation (13) is controlled in galvanostatic mode, while the corresponding PV voltage $v_{PV}\left(t\right)$ serves as the response signal for the impedance calculation according to Equation (15). From the measurements of $i_{PV}\left(t\right)$ and $v_{PV}\left(t\right)$, it is calculated the impedance value for a particular frequency $f_{IS}$. Then, the process is repeated for each frequency to generate the IS.

### 3.2. Duty Cycle Discretization Errors in Digital Implementation

The previous description assumes an ideal duty-cycle modulation, but in practice the sinusoidal perturbation relies on PWM generation, which is inherently limited by the digital resolution of the control hardware. Specifically, the achievable duty-cycle resolution depends on the timer base period (TBPRD) of the digital controller, which is an integer constant that defines the number of discrete clock counts per PWM period, therefore it determines the smallest possible change in the duty cycle. Therefore, the effective duty cycle d[k] at a given time instant *k* is expressed as:

(19) $$d\left[k\right]=\frac{CMPA[k]}{TBPRD}$$

In the previous expression CMPA[k] is the comparison register value, which is an integer variable that represents the number of timer counts during which the PWM output remains active. Since CMPA[k] can only take integer values, the duty cycle is quantized in discrete increments with the following size:

(20) $$\Delta d=\frac{1}{TBPRD}$$

Then, for a desired sinusoidal perturbation of amplitude $d_{IS}$, the effective resolution of the excitation is limited by Δd. If Δd is not sufficiently small compared to $d_{IS}$, the perturbation becomes distorted, introducing harmonic components that reduce the accuracy of the impedance estimation. Moreover, the number of samples per perturbation period is constrained by both the switching $f_{SW}$ and perturbation $f_{IS}$ frequencies as shown in (21).

(21) $$N_{s}=\frac{f_{SW}}{f_{IS}}$$

A sufficiently large $N_{s}$ is required to ensure smooth waveform in the sinusoidal disturbance introduced into d[k]. When $N_{s}$ is too small, the perturbation deviates from its ideal sinusoidal form, which results in the reduction of energy on the main disturbance harmonic ($f_{IS}$) and an increment of energy at the high frequency disturbance harmonics.

Combining those effects, the effective duty cycle applied to the power converter, when it is implemented on an embedded system, can be expressed as shown in (22), where ϵ[k]=Δd/2 represents the average quantization error introduced by the discrete duty-cycle. Minimizing ϵ[k] requires configuring TBPRD to a sufficiently large value, while also ensuring that $f_{IS}$ is chosen such that $N_{s}$ remains high. These conditions guarantee that the applied perturbation follows, as close as possible, the desired sinusoidal reference across the frequency range of interest.

(22) $$d\left[k\right]\approx d_{DC}+d_{IS}\cdot \sin\left(\frac{2\pi f_{IS}k}{f_{SW}}\right)\pm \epsilon \left[k\right]$$

### 3.3. Limitations in Perturbation Amplitude

The previous discrete formulation (22) reveals the second important limitation: the maximum feasible perturbation amplitude. In practice, the duty cycle must remain within its physical bounds,

(23) 0<d[k]<1,∀k,

which imposes the following constraints:

(24) $$0<d_{DC}-d_{IS}-\epsilon \left[k\right]\;\mathrm{and}\;d_{DC}+d_{IS}+\epsilon \left[k\right]<1.$$

Equation (24) shows that the maximum allowable perturbation depends not only on $d_{DC}$ but also on the quantization error ϵ[k]. In the ideal case (ϵ[k]=0), the maximum perturbation amplitude is given in (25).

(25) $$d_{IS,\;max}=\min\left(d_{DC},\;1-d_{DC}\right)$$

Centering the duty cycle around $d_{DC}\approx 0.5$ maximizes this margin because it provides equal margins for upward and downward excursions. Nevertheless, $d_{DC}$ is defined depending on the operation point where the IS measurement is performed. Therefore, it is important to evaluate restriction (25) at each operating point because it will define the maximum amplitude of the sinusoidal disturbance. In general, $d_{IS}$ must remain sufficiently small to satisfy small-signal assumptions, while large enough to provide PV current disturbances that generate voltage responses distinguishable from the measurement noise.

### 3.4. Practical Frequency Limitations for IS Using Power Converters

The selection of the excitation frequencies is fundamentally constrained by the characteristics of the power converter that generates the perturbation and by the measurement hardware that records the PV voltage and current. Although, in theory, sinusoidal perturbations could be generated for any frequency satisfying $0<f_{IS}<\frac{f_{SW}}{2}$ according to the Nyquist–Shannon sampling theorem [11], that range is not realistic for IS measurements performed with a switching power converter.

A first limitation arises from the number of PWM switching cycles available to represent the sinusoidal perturbation. As shown in (21), the number of samples per perturbation period is defined by the ratio between the converter switching frequency ($f_{SW}$) and the excitation frequency ($f_{IS}$). If $N_{s}$ becomes too small, the duty-cycle waveform cannot reproduce a smooth sinusoidal shape, and the resulting perturbation contains significant harmonic distortion. To guarantee an acceptable waveform fidelity, $N_{s}$ must be greater than a minimum value $N_{sm}$, which depends on the resolution of the PWM hardware and the acceptable distortion levels. This condition imposes the following upper bound:

(26) $$f_{IS}<\frac{f_{SW}}{N_{sm}}.$$

As $f_{IS}$ approaches $f_{SW}$, switching-ripple components in $i_{PV}\left(t\right)$ and $v_{PV}\left(t\right)$ increase and become closer to the excitation frequency, further degrading the signal-to-noise ratio and reducing the accuracy of the measured impedance. Thus, the practical upper limit of the usable IS frequency range is typically much lower than $\frac{f_{SW}}{2}$.

On the other hand, the minimum excitation frequency is constrained by the data acquisition system and by the stability of the PV operating point. Very low excitation frequencies require long acquisition times, which may be incompatible with the sampling capabilities of the controller or may lead to variations in irradiance or temperature during the measurement interval. As a result, the minimum usable frequency, denoted as $f_{min}$, depends on the specific experimental setup and may range from the millihertz to the hertz domain, depending on sensor bandwidth, data logging rate, and environmental stability.

Considering both bounds, the practical IS frequency range when measurements are performed through a DC-DC converter can be expressed as:

(27) $$f_{min}<f_{IS}<\frac{f_{SW}}{N_{sm}}$$

This restriction highlights that, although the converter enables perturbation injection and IS calculation, the achievable frequency range is limited by switching dynamics, PWM discretization, measurement noise, and the stability of the operating conditions.

## 4. Simulation Results

**Figure 5:**
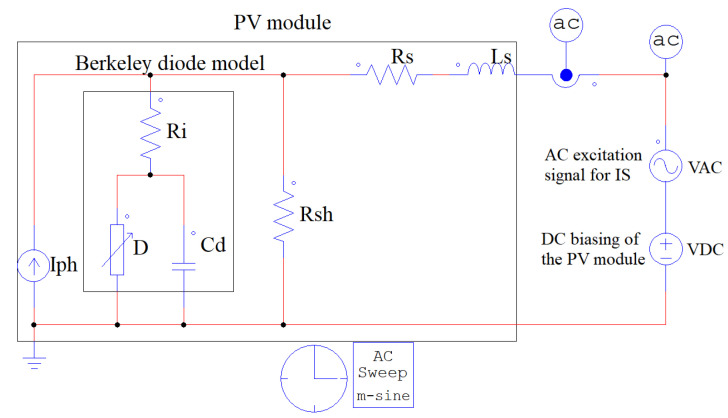
PSIM simulation setup of the proposed IS reference system.

The proposed system is validated by using PSIM software (version 2025.1) to compare the IS measurement obtained with the power converter and a reference IS obtained with the AC-sweep analysis included in the simulator. The PV module SOLBIAN Flex-SP50L (Solbian Solar, Graz, Austria) [23] is selected for this study because its static and dynamic behaviors, modeled using the SDM with the Berkeley diode, have been thoroughly validated for both the I-V curve reproduction and impedance spectroscopy applications [13,24,25,26]. Figure 5 shows the reference IS simulation setup, where the PV module is represented by the SDM with the diode modeled by the Berkeley representation. In this model, the elements Iph, Rsh, and Rs correspond to the components of the SDM model, while Ls corresponds to the parasitic inductances of the PV module. The Berkeley representation parameters are Ri, the diffusion capacitance Cd, and the diode *D*, which is implemented with a nonlinear element that includes the Shockley diode equation. The DC voltage (VDC) sets the operating point of the circuit, while a small-signal perturbation is applied through the AC voltage source (VAC), which is controlled by the AC Sweep m-sine block. The AC measurement probes are used to capture the voltage and current responses of the PV model. The AC-Sweep is configured with the frequency range [10 Hz, 20 kHz] to meet the frequency range adopted in the experiments reported in [13].

**Figure 6:**
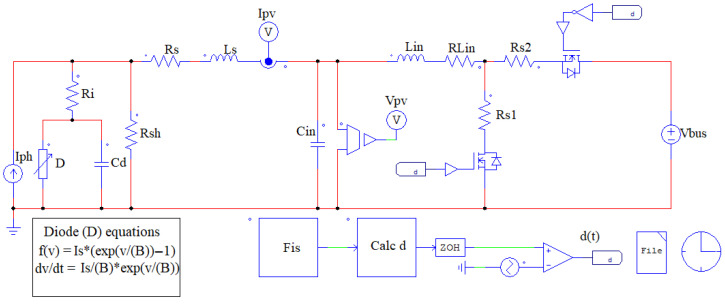
PSIM simulation setup of the proposed system for IS using a power converter.

Then, the proposed structure is implemented by connecting the same PV module equivalent circuit to a synchronous Boost converter and a DC bus as it is shown in Figure 6. In this circuit, Lin and Cin are the converter inductance and capacitance, Rs1, Rs2, and RLin are the resistances of the MOSFETs and the inductor, and Vbus is the DC bus voltage. Moreover, the block labeled “Fis” defines the frequency at which the IS is evaluated. The block labeled “Calc d” calculates the duty cycle as $d\left(t\right)=d_{DC}+d_{IS}\;sin\left(wt\right)$ according to (5), where $d_{DC}$ is a constant defined to fix the operating point, and $d_{IS}$ is calculated by using (12) to impose the desired amplitude in the sinusoidal component of $i_{PV}$ (i.e., $i_{IS}$). Moreover, a Zero Order Hold (ZOH) block is included to reproduce the discretization error of the real implementation. Such a ZOH block is configured with a sampling frequency equal to the converter’s switching frequency ($f_{SW}$). Finally, the data for the impedance calculation are obtained from the sensors labeled “Ipv” and “Vpv” in Figure 6, which are processed in MATLAB to generate the IS.

The parameters used for the reference simulation and for the proposed system are summarized in Table 1. The PV model parameters correspond to an operating condition defined by $V_{Q}=8.63\;V$ and $I_{Q}=1.75\;A$, under an irradiance of $700\;{W/m}^{2}$ and a module temperature of 324.15K, which corresponds to the experimental conditions reported in [13]. Table 1 also includes the definition of *D*, which represents a nonlinear element modeled by the Shockley Equation (1), with parameters $\left(I_{\mathrm{sat}},\eta ,T\right)$ and the fundamental constants *q* and *k*. It is important to note that real photovoltaic devices could not exhibit the ideal capacitive behavior assumed for the diffusion capacitance $C_{d}$, since distributed recombination and charge-transport phenomena often introduce non-ideal effects [27,28]. Nevertheless, the value adopted in this work is taken from previous impedance spectroscopy studies reported for the same PV module [13], providing a consistent and experimentally grounded starting point for the proposed modeling and validation.

**Figure 7:**
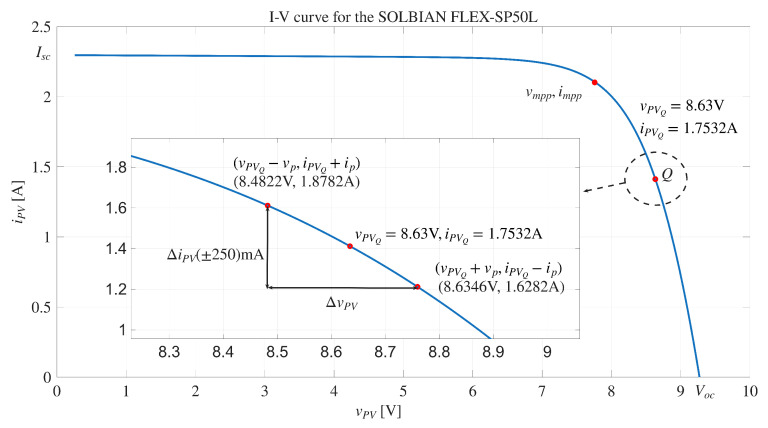
I-V curve and operating point of the PV module SOLBIAN Flex-SP50P for an irradiance of of 700 W/m^2^ and a PV module temperature of 324.15 K.

Figure 7 reports the I-V curve of the PV module and the operating point where the IS is measured, which is obtained by performing a voltage sweep at the terminals of the equivalent circuit that models the PV module. This figure also illustrates the amplitude defined for the current disturbance ($|I_{PV}\left(j\omega \right)|$ = 125 mA).

**Figure 8:**
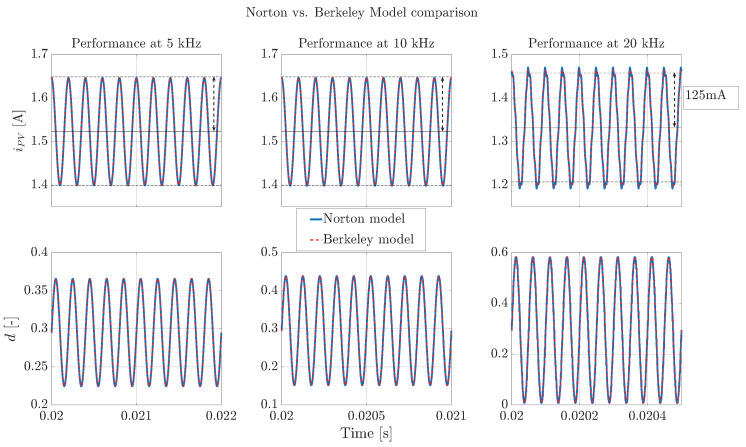
Performance comparison between the Norton and Berkeley models for impedance spectroscopy at a given operating point.

The proposed solution defines the duty cycle perturbation using expression (12), which is derived considering the Norton small-signal model. While the Berkeley model is adopted to represent the module’s nonlinear static and dynamic behavior, the perturbation generation is formulated based on the linearized dynamics around the operating point. The validity of the Norton model around a given operation point is confirmed in Figure 8, where the current waveform imposed by the converter to each model, using the duty cycle perturbation (12), is simulated at three different frequencies (5 kHz, 10 kHz, 20 kHz). Those results show that the Norton model, linearized under the same bias condition, reproduces the Berkeley model behavior in small-signal, thus confirming the validity of the perturbation equations obtained for IS tests.

**Figure 9:**
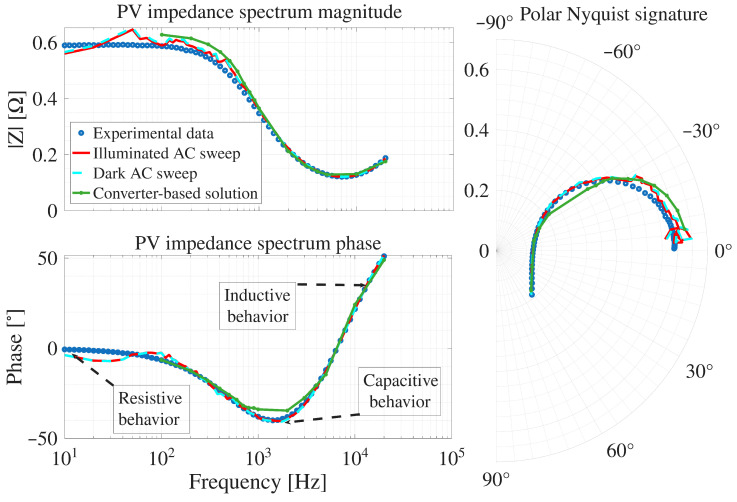
Comparison of IS spectra from the proposed system, AC sweep, and experimental data of the SOLBIAN Flex-SP50P.

Then, the proposed technique is evaluated using the more complete Berkeley model. The resulting IS measurements obtained with both the simulator AC-sweep and the converter-based solution, along with the experimental reference data from [13], are presented in Figure 9. Such a simulation reports the AC-sweep results under both illuminated (red data) and dark conditions (light blue dashed data), which are superimposed because, although the simulated irradiance is different, both simulations are performed at the same operating point, resulting in nearly identical dynamic behavior and IS spectra. The converter-based solution (green data) closely matches the AC-sweep results (red and light blue dashed data), where minor deviations are observed at lower frequencies (f<1kHz) in the magnitude and at medium frequencies (0.8kHz<f<3kHz) in the phase, indicating small discrepancies in the low- and mid-frequency ranges. Finally, the experimental data (blue dots data) confirms the accuracy of all simulated IS, i.e. both illuminated and dark Berkeley models and the converter-based solution.

From Figure 9, the sign change in the phase angle is a consequence of the different physical phenomena dominating the PV module impedance across the analyzed frequency range. At low frequencies (below 50Hz), the phase remains close to zero, indicating a predominantly resistive behavior. In this region, the response is mainly governed by the steady-state conduction mechanisms and ohmic resistances of the PV module. At intermediate frequencies (between 50Hz and 6kHz), the phase becomes negative, revealing a predominantly capacitive impedance. This behavior is associated with charge storage effects, including the junction capacitance of the PV cells and the diffusion capacitance of the diode, which dominate the dynamic response in this frequency range. At higher frequencies, above 6kHz, the phase shifts toward positive values, indicating an inductive behavior. This regime is mainly driven by parasitic inductances inherent to the PV module interconnections, bonding wires, and external wiring, which increasingly influence the impedance as frequency rises. Therefore, the transition from resistive to capacitive behavior, and finally to inductive behavior, across the frequency spectrum explains the occurrence of both positive and negative phase angles in the measured IS, reflecting the different dominant physical mechanisms at each frequency range.

To quantify the deviation between the simulated and experimental IS, the Range-Average Absolute Error (RAAE) metric is used. This metric, defined in (28), normalizes the absolute error by the dynamic range of the reference signal, providing a dimensionless measure of relative accuracy that remains consistent across different magnitudes and scales [29]. In such an equation, the numerator corresponds to the average absolute error between the simulated and experimental samples, while the denominator max(y)−min(y) defines the dynamic range of the reference signal used for normalization. Here, $y_{i}$ represents the experimental value at sample *i*, ${\hat{y}}_{i}$ is the corresponding simulated value, and max(y)−min(y) denotes the dynamic range of the reference signal. In this work, the RAAE is expressed as a percentage to facilitate comparison between magnitude and phase errors. The use of RAAE is particularly suitable when evaluating errors near zero-valued measurements, where traditional error metrics such as RMSE can produce disproportionately large values due to normalization by small denominators.

(28) $$\mathrm{RAAE}\left(\%\right)=100\times \frac{\frac{1}{N}\sum_{i=1}^{N}\left|y_{i}-{\hat{y}}_{i}\right|}{\max(y)-\min(y)}$$

**Figure 10:**
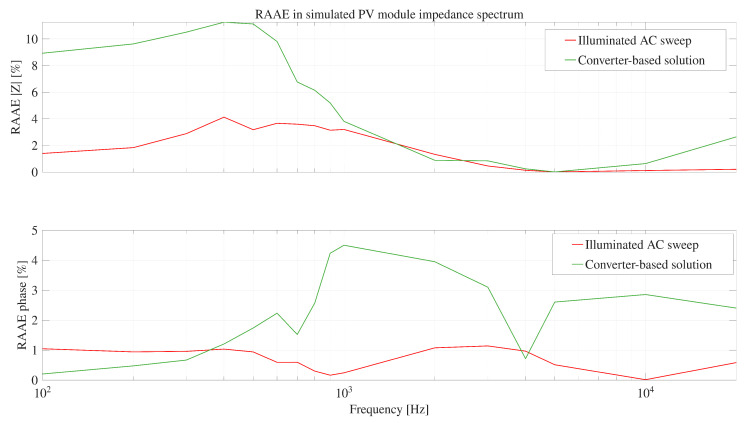
RAAE-based comparison of magnitude and phase deviations for the proposed system and the AC sweep against the experimental reference data.

The RAAE for the IS obtained with the simulator AC-seep and the proposed system, against the experimental reference data, are reported in Figure 10. It can be observed that the RAAE corresponding to the simulator AC sweep shows an error lower than 4%; while, the error with the proposed system reaches 11.2% at low frequencies; however, at higher frequencies, the errors are similar. Therefore, the simulation results show that the proposed system is able to reproduce the experimental IS by using the power converter to generate the sinusoidal disturbances.

## 5. Experimental Results

**Figure 11:**
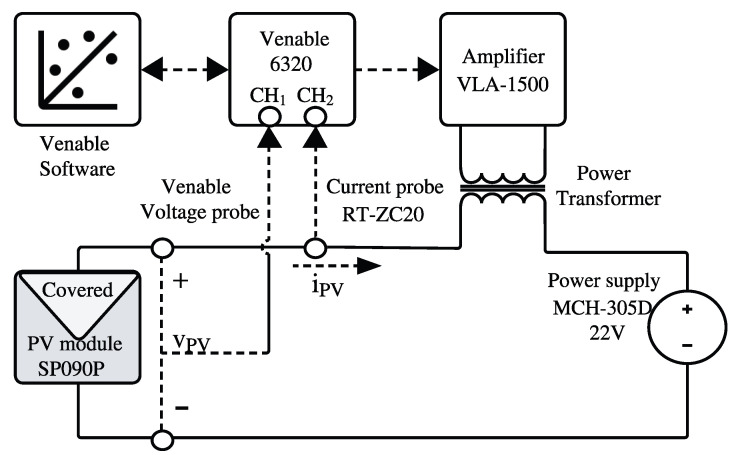
Proposed structure for the reference experimental setup.

The proposed solution was validated using two experimental setups. The first one is used to obtain the reference IS of a PV module using a commercial FRA. The second one implements the proposed solution for IS measuring using a Boost converter. The SP090P PV module (SolarTech, Hawkesbury, ON, Canada) [30] was selected for those experiments due to its availability and its compatibility with the power and voltage ratings of the implemented converter, ensuring safe operation and reliable experimental validation. The first setup is formed by the PV module, a MCH-305D power supply (MCH, Shenzhen, China) [31], a Rogowski current probe RT-ZC20 (Rohde & Schwarz, Munich, Germany) [32], and a Venable 6320 FRA (Venable Instruments, Austin, TX, USA) [33] with a VLA 1500 power amplifier and a high-frequency transformer [34]. It is worth mentioning that, even though the Venable 6320 maximum frequency is in the MHz range, when it is connected to the power amplifier and the high-frequency transformer, such a frequency range is limited to 30 Hz–250 kHz. The connections of the elements in the first setup are presented in Figure 11. The Venable FRA controls the associated amplifier and power transformer to produce the IS excitation signal, while the power supply sets the operating point of the PV module. Finally, the FRA captures the IS data to be stored in a computer.

**Figure 12:**
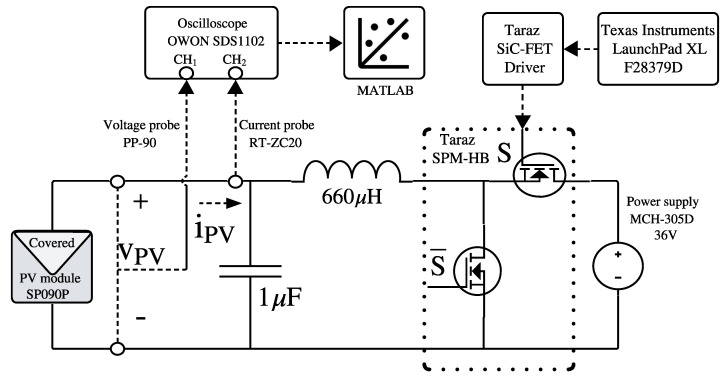
Experimental setup to test the proposed solution.

The second experiment consists of a synchronous Boost converter with an L-C filter of 660 μH and 1 μF, respectively, implemented using the 1.2 kW half-bridge module SPM-HB (Taraz Technologies, Rawalpindi, Pakistan) [35]. This setup uses the same PV module, current probe (for the oscilloscope), and power supply present in the first experiment. A Texas Instruments F28379D LaunchPad XL microcontroller development kit [36] provides the control signals, while measurements are recorded using an OWON SDS1102 oscilloscope (OWON, Zhangzhou, China) [37] equipped with a 1× voltage probe PP-90 [38]. The interconnection of all components in this second setup is shown in Figure 12. The microcontroller generates a PWM signal at 200 kHz (the maximum switching frequency supported by the half-bridge module), with the duty cycle modulated according to (5), thus producing a constant duty cycle superimposed with a sinusoidal disturbance of amplitude $d_{IS}$. For the experiments, the amplitude of the sinusoidal disturbance in the PV module current ($|I_{PV}\left(j\omega \right)|$) was set to 200 mA to ensure a sufficiently high signal-to-noise ratio relative to the electronic noise present in the measurements. The experimental frequency range considered in this test extends from 100 Hz to 20 kHz, which is defined by the limitations of the reference measurement setup and the constraints imposed by the converter-based implementation. The reference system incorporates a high-frequency transformer whose bandwidth confines the measurable impedance spectrum to 30 Hz–250 kHz. Moreover, the proposed power converter operates at a switching frequency of 200 kHz. Then, to maintain adequate separation between the injected perturbation and the switching-related harmonics, the excitation frequency is limited to values below the switching frequency, typically to about one decade lower, i.e., below 20 kHz. At the lower-frequency end, excitation frequencies below 100 Hz are avoided because slow system dynamics can induce variations in the operating point during the measurement due to variations due to variations in the irradiance.

To evaluate the accuracy of the IS measured with the proposed system, it is necessary to perform the measurements with both the FRA and the power converter under identical operating conditions. Therefore, the experiments must be done under the same irradiance and temperature, and at the same operating point $\left(V_{Q}=21.2\;V,I_{Q}=1\;A\right)$ on the right-side of the PV module’s I-V curve. This is critical for the experimental comparison, because different operating conditions or operating points produce different IS data, making it impossible to accurately quantify the measurement errors of the proposed approach.

**Figure 13:**
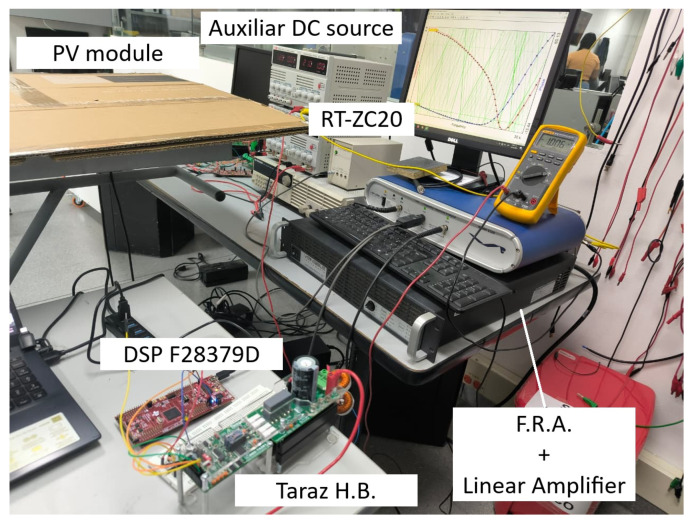
Photo of the two experimental setups used for the experimental results.

Ensuring consistent outdoor conditions (irradiance and ambient temperature) for the PV module is very difficult, hence the experimental measurements were carried out inside the laboratory and completely covering the PV module with a cardboard sheet (i.e., dark conditions) as shown in Figure 13. In this way it is possible to guarantee the same irradiance (0 W/$m^{2}$) and temperature of the PV module (controlled laboratory temperature of 22 °C) for the IS measurements with both the the FRA and the proposed system. Under those conditions $I_{ph}=0$ A and the I-V curve of the PV module is located in the fourth quadrant. Nevertheless, the shape of the I-V curve is the same that in the first quadrant; hence, the operating point $\left(V_{Q},I_{Q}\right)$ can be defined by injecting a current to the PV module ($i_{DC}<0$ A), while the PV module voltage remains positive ($v_{DC}>0$ V) [39]. For the experiments, the PV module temperature was 300.15 K (27 °C).

**Figure 14:**
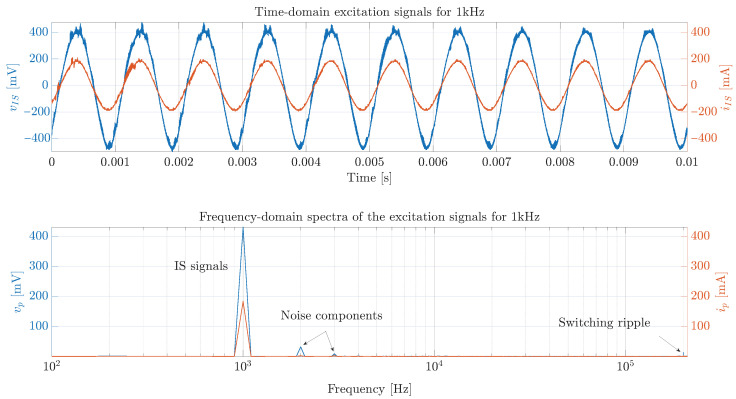
Example of voltage and current measurements obtained with the proposed system for a disturbance of 1 kHz.

The PV module voltage and current measurements for a disturbance of 1 kHz–6283.19 rad/s (low frequency) are presented in Figure 14, which is an example of the current disturbance produced with the power converter. This figure shows that the amplitude of the current disturbance (red waveform) is 200 mA as expected; however, the noise in the voltage measurement (blue waveform) makes it difficult to determine the amplitude and phase with respect to the current. Therefore, the FFT is applied to the current and voltage signals (lower traces of Figure 14) to obtain the PV module impedance magnitude and phase: $|Z_{PV}\left(j\omega \right)|=2.34\;\Omega$ and $∠Z_{PV}\left(j\omega \right)=-4.242$°.

**Figure 15:**
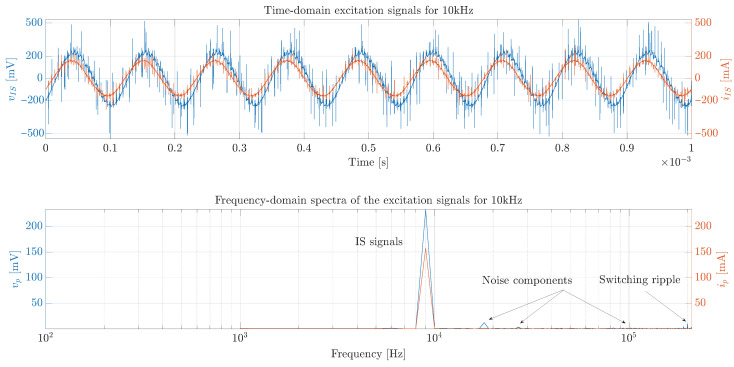
Example of voltage and current measurements obtained with the proposed system for a disturbance of 10 kHz.

Another example of The PV module voltage and current measurements for a disturbance of 10 kHz–62,831.85 rad/s (high frequency) is presented in Figure 15. In this case, the current disturbance amplitude remains approximately constant, but there is a significant attenuation of the amplitude in the sinusoidal response of the PV module voltage, which makes difficult the estimation of the impedance amplitude and phase. For this case, the impedance’s magnitude and phase obtained after applying the FFT (lower traces of the figure) are $|Z_{PV}\left(j\omega \right)|=1.37\;\Omega$ and $∠Z_{PV}\left(j\omega \right)=-16.4219$°.

**Figure 16:**
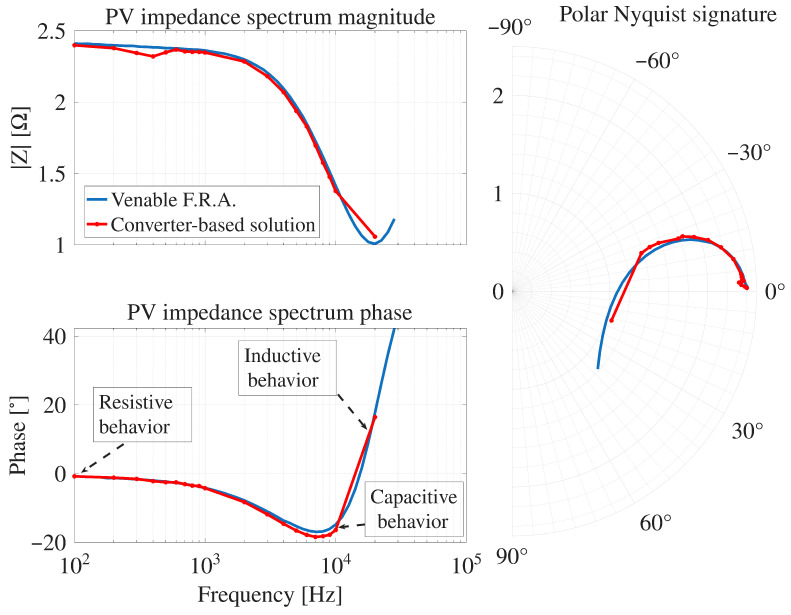
Comparison of IS spectra from the Converter-based solution and Venable F.R.A. of the SP090 PV module.

By repeating the previous procedure for 20 different frequencies it is obtained the IS with the proposed converter-based system. Figure 16 shows the experimental IS obtained with both the commercial FRA and the proposed technique.

The experimental results shown in Figure 16 exhibit a frequency-dependent behavior consistent with the expected impedance characteristics of the PV module. At the lowest frequencies, the response is predominantly resistive; however, since the experimental sweep starts at 100Hz, this regime is only partially captured. Over most of the analyzed spectrum, the phase becomes negative, indicating a mainly capacitive behavior, which persists up to approximately 11kHz. Beyond this frequency, the phase shifts toward positive values, revealing the increasing dominance of inductive effects at higher frequencies.

**Figure 17:**
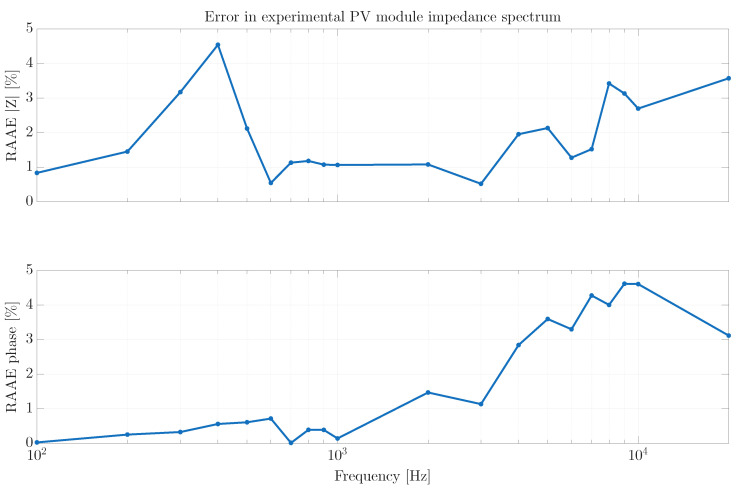
Impedance Spectrum error: proposed solution vs. commercial FRA.

Those results show that the IS obtained with the proposed system exhibits the expected behavior across the evaluated frequency range, thus it is very similar to the IS data generated by the commercial Venable FRA. Such a correct behavior of the developed converter-based system is confirmed by the RAAE plot presented in Figure 17. For the magnitude and phase estimations the RAAE are below 4.6% for all the frequencies, where the biggest magnitude error occurs around 400 Hz (error of 4.6%), and at high frequency the biggest error occurs at 20 kHz (3.8%). For the phase estimation the biggest errors (4.7%) are located at high frequencies (around 10 kHz). Finally, the average magnitude RAAE is 1.92% with a standard deviation of 1.14%, and the average phase RAAE is 1.82% with a standard deviation of 1.74%. Those results put into evidence the satisfactory performance of the IS measurement performed with the proposed solution.

In general, the errors in the magnitude and phase estimation are produced by two main reasons. The first one is the limited resolution to generate the sinusoidal perturbation signal in the duty cycle with a microcontroller, as explained in Section 3. The second reason is the switching noise introduced by the power converter in the measurement of the PV module’s voltage and current, which increments for high frequencies of the sinusoidal disturbance; this was illustrated in Figure 14 and Figure 15.

From an economic perspective, the proposed system represents a highly cost-effective alternative to conventional frequency-response devices. A commercial Venable FRA setup, including a linear amplifier, power transformer, a current probe, and dedicated software, typically costs around $48,300 USD. In contrast, the proposed configuration based on a half-bridge converter with SiC FETs has an estimated total cost of approximately $9000 USD, including the oscilloscope, the current probe, and the auxiliary wiring and measurement accessories. This results in a reduction of nearly six times in implementation cost. However, considering that oscilloscopes and current probes are standard equipment in any power electronics laboratory, the proposed solution only requires the power converter and the auxiliary wiring with an estimated cost of $400 USD, thus resulting in a reduction of nearly one hundred twenty times in implementation cost, demonstrating that high-cost instrumentation is not strictly required to achieve reliable IS measurement of a PV module.

Despite the significantly lower cost, the proposed system provides an acceptable level of accuracy, with deviations below 4.6% when compared to a commercial FRA one hundred twenty times more costly. Furthermore, its ability to perform online IS measurements directly on the power converter enables in-field diagnostic of photovoltaic generators and applications that uses power converters (e.g., batteries and wind turbines), particularly in remote or industrial environments where access for specialized personnel is limited. This balance between cost, accuracy, and operational flexibility highlights the potential of the proposed approach as a practical and scalable alternative for real-time impedance spectroscopy in power electronics systems.

## 6. Conclusions

This work presented a complete methodology for performing IS on PV modules using a power converter as both the excitation and measurement interface. The study included the theoretical modeling of the converter–PV system, the derivation of the required duty-cycle perturbation to guarantee a constant excitation magnitude across frequencies, and the implementation of a converter-based IS setup. A commercial FRA was used as a reference to enable a fair comparison between measurement approaches.

The main contributions of this work are identified. First, an analytical relationship between the duty-cycle perturbation and the resulting current excitation amplitude at the PV terminals was derived, enabling frequency-adaptive perturbation synthesis without additional hardware. Second, a practical methodology to perform IS using a standard power converter was developed, requiring only a microcontroller to generate the sinusoidal perturbation and an oscilloscope to acquire the voltage and current signals. Third, the proposed system provides a low-cost and scalable alternative to conventional FRA-based instrumentation, thereby enabling IS capabilities directly integrated into power electronics interfaces.

The proposed methodology was validated using simulation and experimental measurements under controlled dark conditions, which ensures repeatability and identical operating points. The experimental results confirmed that the converter-based IS accurately replicates the impedance magnitude and phase obtained with the commercial FRA across the evaluated frequency range. The relative amplitude and phase errors (RAAE) remained below 4.6% for all frequencies, with the largest deviations occurring at medium and high frequencies due to quantization limits in the duty-cycle generation and switching noise in the converter. These results confirm that the proposed approach provides the fidelity required for reliable PV impedance characterization.

From an economic perspective, the converter-based IS system achieves a cost reduction of nearly six times compared to a commercial FRA setup if the oscilloscope must me also acquired, and a reduction of nearly one hundred twenty times if the oscilloscope is already available, thus demonstrating that high-cost instrumentation is not necessary to obtain accurate PV impedance spectra. This cost advantage, combined with the ability to integrate IS functionality directly into the converter hardware, highlights the potential of this method for in-field diagnostics, condition monitoring, and real-time health assessment of PV generators and other energy conversion systems.

Future work will explore the extension of the proposed impedance spectroscopy methodology to PV technologies exhibiting pronounced current-voltage hysteresis, such as perovskite solar cells. This behavior complicates the definition of a unique operating point and the extraction of the maximum power point, and may require adapting the measurement procedure using operating-point stabilization or hysteresis-aware modeling. Addressing those aspects would broaden the applicability of the proposed converter-based approach to emerging PV technologies.

## Figures and Tables

**Table 1:** Parameters used for the simulation of the proposed system and the reference IS circuit.

Parameter	Value	Parameter	Value
Iph	2.7461 A	D	Shockley Equation (1)
Cd	44.88 mF	Ri	52.7 mΩ
Rsh	408.30 Ω	Rs	62.8 mΩ
Ls	1.3043 μH	Cin	1 μF
Lin	110 μH	RLin	10 mH
Rs1 = Rs2	75 mΩ	Vbus	12 V
η	1.3	Fsw	1 MHz
Is	25.50 nA	T	324.15 K

## Data Availability

All data used in this study are reported into the manuscript.
